# Evaluation of key miRNAs during early pregnancy in Kazakh horse using RNA sequencing

**DOI:** 10.7717/peerj.10796

**Published:** 2021-02-23

**Authors:** LingLing Liu, Chao Fang, YinZe Sun, WuJun Liu

**Affiliations:** 1 College of Animal Science, Xinjiang Agriculture University, Urumqi, Xinjiang, China; 2 Department of Animal Production, Farah Research Centre from the Faculty of Veterinary Medicine, University of Liège, Liège, Belgium

**Keywords:** Kazakh horse, miRNAs, Milk yield, High-throughput sequencing

## Abstract

**Background:**

miRNA has an important role in cell differentiation, biological development, and physiology. Milk production is an important quantitative trait in livestock and miRNA plays a role in the amount of milk produced.

**Methods:**

The role of regulatory miRNAs involved in equine milk production is not fully understood. We constructed two miRNA libraries for Kazakh horse milk production from higher-producing (H group) and lower-producing (L group) individuals, and used RNA-Seq technology to identify the differentially expressed miRNAs between the two milk phenotypes of Kazakh horses.

**Results:**

A total of 341 known and 333 novel miRNAs were detected from the H and L groups, respectively. Eighty-three differentially expressed miRNAs were identified between the H and L group s, of which 32 were known miRNAs (27 were up-regulated, five were down-regulated) and 51 were novel miRNAs (nine were up-regulated, 42 were down-regulated). A total of 2,415 genes were identified. The Gene Ontology (GO) and Kyoto Encyclopedia of Genes and Genomes (KEGG) pathway analyses showed that these genes were annotated to mammary gland development, mammary gland morphogenesis, tissue development and PI3K-Akt signaling pathways, insulin signaling pathway and TGF-beta signaling pathway, among others. Five miRNAs (miR-199a-3p, miR143, miR145, miR221, miR486-5p) were identified as affecting horse milk production and these five miRNAs were validated using qRT-PCR.

**Conclusions:**

We described a methodology for the transcriptome-wide profiling of miRNAs in milk, which may help the design of new intervention strategies to improve the milk yield of Kazakh horses.

## Introduction

Mare’s milk and its byproducts have a unique nutritional profile. They contain nutrients that fight fatigue and can alleviate physical exhaustion (Liu et al., 2010). However, it is difficult to determine which horses will produce a high milk yield. We previously used selection signals to screen for key genes related to the high milk production in Kazakh horses. In this study we screened miRNAs of high-yielding horses at the transcriptome level to provide markers for the selection of Kazakh horses with high milk production.

A variety of RNA sources, including milk somatic cells (MSCs) (Wickramasinghe et al., 2012), mammary gland biopsies (Lemay et al., 2013) and milk fat globules (Maningat et al., 2009), have been used to study the transcriptome of the mammary gland during lactation. For this study, RNA was extracted from equine milk fat globules. Mammary epithelial cells (MECs) are essentially biofactories of lipids, proteins, and carbohydrates for milk during lactation (Lemay et al., 2007). Milk fat is secreted via a budding (apocrine) mechanism and can carry with it a crescent of the MEC cytoplasm enveloped in plasma membrane (Huston & Patton, 1990). A total of 1 to 38% of milk fat globules (MFG) contain these crescents (Patton & Huston, 1988), which allow us to use milk to analyze the miRNA.

miRNA in the mammary gland can regulate post-transcriptional expression of target genes and thereby control the formation of mammary ducts and acini, as well as the proliferation, differentiation, and apoptosis of mammary gland epithelial cells. These activities influence mammary gland development and lactation (Ji et al., 2013; Ji et al., 2015; Ji et al., 2019).

miRNAs represent a class of small (20 nt–25 nt) noncoding RNA, which regulate gene expression by binding to specific mRNA targets and promoting their degradation and/or translational inhibition (Baek et al., 2008). High-throughput sequencing provides a unique opportunity to catalog the RNA and study miRNA-mRNA interactions in mammary tissue. Wang et al. (2018) showed that miRNAs may play an important role in the regulation of milk quality and milk secretion in the process of mammary gland differentiation (Wang et al., 2018). Mobuchon et al. (2015) conducted a major identification study on miRNA expressed in the goat mammary gland at the peak of lactation.

Many recent studies have focused on horses and the expression of plasma miRNAs, which show sequence and nucleotide composition differently than those found in non-plasma miRNAs (Lee et al., 2016). Cappelli et al. (2008) identified some ci-miRNAs that revealed responses to endurance exercise (Cappelli et al., 2008). Cowled et al. (2017) identified thirty-seven miRNAs that were differentially expressed in horses infected with Hendra virus. McGivney et al. (2017) identified plasma ci-miRNA profiles and skeletal muscle miRNAs before and after exercise in Thoroughbreds to evaluate for the presence and effect of hemolysis on plasma ci-miRNA. However, there have been no reports on transcriptome sequencing using horse milk. To date there has only been one study evaluating miRNA in pregnant horses (Loux et al., 2017).

To the best of our knowledge, there has been no previous research on the expression of miRNAs in milk fat from the Kazakh horse. We determined miRNA expression in horses with high milk yields and compared them to horses with low milk yields.

## Materials and Methods

### Ethics statement

All experimental procedures involving animals were approved by the Animal Care and Use Committee of Xinjiang Agricultural University, Urumqi, Xinjiang, China (animal protocol number: 2017008).

### Animals and sample collection

All experiments were performed on eight healthy Kazakh horses. Eight lactating horses, parity four, were selected from Fuyun County. Lactating horses were allocated to the H group (milk yield >  7 kg) or the L group (milk yield <  3 kg) according to their milk yield (Table S1). A 300 mL sample of milk was collected from each horse, quickly frozen on liquid nitrogen, and brought back to the laboratory where the samples were kept at −80 °C until testing.

### Milk fat extraction

A total of 50 mL (2700 g) samples of milk were centrifuged at 10 °C for 10 min; the upper layer of milk fat was removed and trizol was added to a 2:8 ratio. The mixture was thoroughly shaken and then frozen at −80 °C.

### RNA extraction

Total RNA extraction was performed using the miRNeasy Mini Kit (217004, QIAGEN, Germany) according to the standard operating procedures provided by the manufacturer. The Qubit^®^2.0 Fluorometer (Life Technologies, Carlsbad, CA, USA) and the Nanodrop One spectrophotometer (Thermo Fisher Scientific Inc., Waltham, MA, USA) were used to assess the concentration and quality of RNA. The integrity of the total RNA was assessed using the Agilent 2100 Bioanalyzer (Agilent Technologies Inc., USA) and samples with RNA integrity number (RIN) values above 7.0 were used for sequencing.

### Small RNA library construction and sequencing

A Qiagen™ Small RNA Library Kit (Qiagen) was used to prepare small RNA-seq libraries according to the manufacturer’s protocol. The Agilent 2100 Bioanalyzer and an Agilent High Sensitivity DNA chip (Agilent Technologies) were used to assess the miRNA-seq libraries. The final product was a library of approximately 173–181 bp. The sequence tags were obtained via Illumina Nova Seq 6000 by the Shanghai Sinomics Corporation.

### Small RNA-seq data analysis

MiRDeep software was used to predict the known and novel miRNAs (Friedländer et al., 2008). The reads were trimmed with Cutadapt v 1.9.1 (Martin, 2011). Reads shorter than 15 bp or longer than 40 bp were discarded. Quality control of the reads was performed using seqtk (1.0-r82-dirty) and R (3.4.2). The ends of the filtered reads were further trimmed to remove low-quality bases (Phred score ≤ 20). The clean reads were mapped to the horse genome (EquCab 2.0) using bowtie (1.0.0). Gene abundance was expressed as counts of exon model per million mapped reads (CPM). R (3.4.2) was used with a self-written script and other software was used with default parameters.

### Identification of differentially expressed miRNAs (DEMs) and prediction analysis of DEMs

The DESeq R package (v. 1.8.3) was used to identify DEMs based on miRNA expression values by pairwise comparison to understand the functional role of DEMs in horse milk of the two horse groups (Wang et al., 2010). The cut-off criteria for DEMs were —log2(Fold change) — ≥ 1 and *P*-value ≤ 0.05. The target genes of these miRNAs were predicted using the miRanda (Betel et al., 2008) analytical tools. MiRNA target gene searching was performed using the MultimiR package, and the search consisted of predicted and validated target genes.

### Enrichment analyses for DEM targets

GO annotations of differentially expressed miRNA target genes were performed using the gene ontology (GO) database (http://geneontology.org/); pathway analysis of differentially expressed genes was performed using the KEGG (Kyoto Encyclopedia of genes and genomes) database. Both the GO annotation and the KEGG pathway analysis had a *P* value of less than 0.05 as a significant enrichment criterion.

### Validation of miRNA expression by real-time PCR (RT-PCR)

Libraries of cDNA were created using the HiScript II Q RT SuperMix. A total of 500 ng RNA was used per reaction, following manufacturer’s instructions. We used 10 µL 2 ×miRNA RT Reaction Buffer, 2 µL miRNA RT Mix with nuclease-free water added to bring the final volume to 20 µL. An additional aliquot was made with water in place of the RNA for a negative control. Tubes were held at 42 °C for 60 min followed by 3 min at 95 °C, then cooled to 4 °C and held at this temperature until use.

Reactions were immediately prepared for qPCR using the miRcute Plus miRNA qPCR Kit (Qiagen). Each reaction consisted of 5 *μ*L 2 ×miRcute Plus miRNA Premix, 1 µL Amplification Primer (2 µM), 1 µL Reverse Primer (2 µM), 2 µL nuclease-free H_2_O, and 1 µL cDNA. All reactions were performed in duplicate. The RNA-free reverse transcription reaction and a cDNA-free PCR reaction were used as negative controls.

Thermal cycling took place on a 7900 real-time qPCR system (ABI, USA), and consisted of an initial incubation at 50 °C for two min, 95 °C for 10 min, 95 °C for 15 s followed by 40 cycles at 60 °C for 1 min, 95 °C for 15 s, 60 °C for 15 s and 95 °C for 15 s. Porcine U6 snRNA (horse) and eca-miR-26a were used as an endogenous reference. Data were normalized by ΔCt = Ct (target) −Ct (mean of sample). The primer sequence of 5 miRNAs are shown in Table S2.

## Results

### Overview of miRNA sequencing

**Figure 1:**
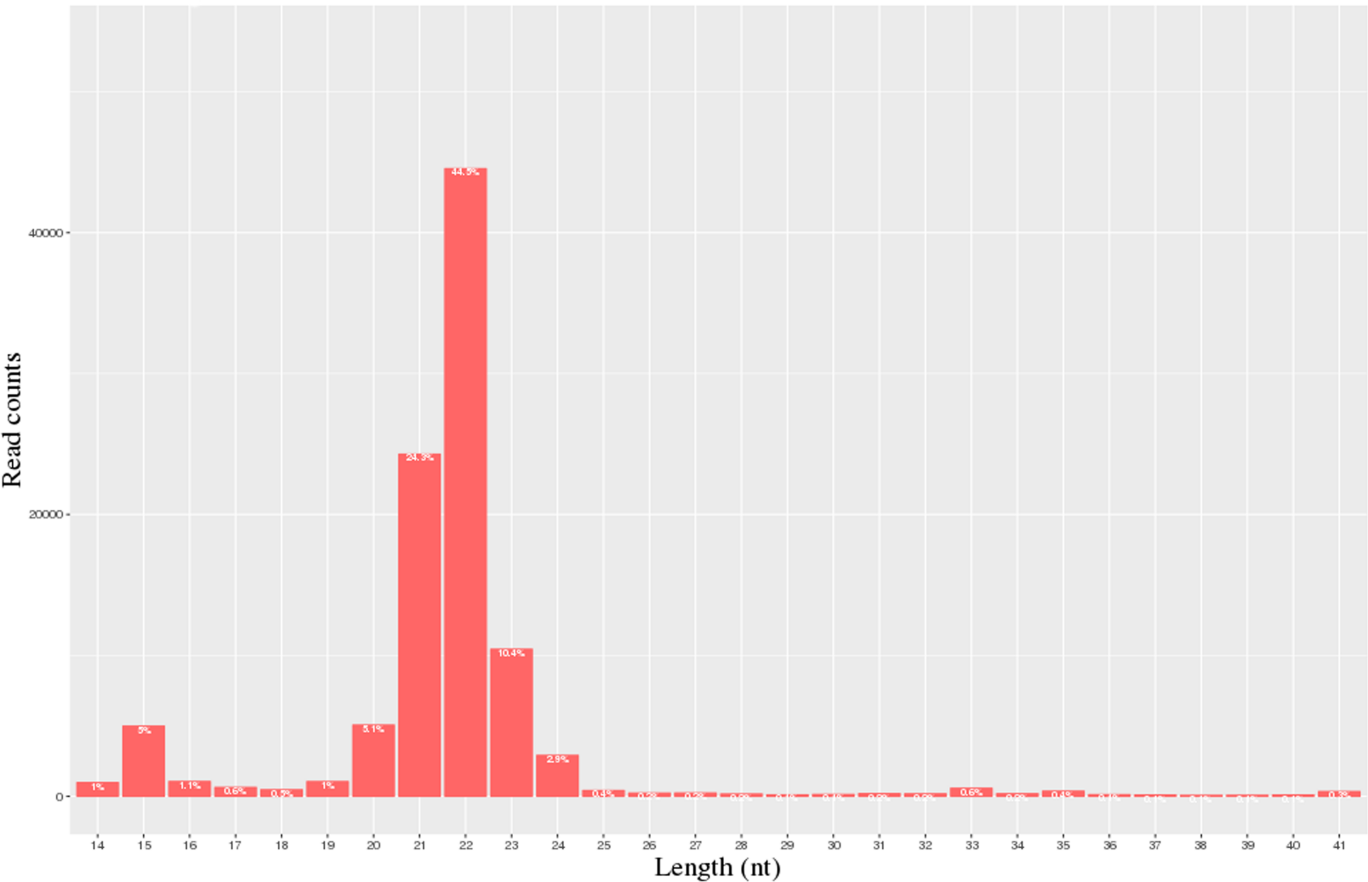
Statistics of miRNA length distribution in H group and L group of horse libraries.

A total of 40.3 million and 28.1 million raw reads were obtained from the milk of the H group and L group, respectively. 28,055,021 and 20,692,008 clean reads from the H and L horse milk libraries, respectively, were selected for further analysis after the reads were assessed for quality (Table 1). The QC results are shown in Table S3. Most of these miRNAs ranged from 20-24 nt, and the read-length distributions of the H group and L groups are shown in Fig. 1.

**Table 1:** Number of clean reads generated from each group and mapping statistics.

Sample	Total reads	Clean reads	Reads mapped	Mapped rate(%)
L1	36,699,449	23,543,739	20,212,101	85.85%
L2	43,485,569	30,563,782	25,563,485	83.64%
L3	46,154,969	33,169,894	25,742,016	77.61%
L4	34,676,651	24,942,671	16,974,152	68.05%
H1	28,025,537	22,618,513	21,571,539	95.37%
H2	27,361,219	19,211,386	16,853,418	87.73%
H3	29,331,228	20,375,830	17,461,553	85.70%
H4	27,732,956	20,562,306	19,133,640	93.05%

We identified 341 miRNAs in the H group and 333 miRNAs in the L group. The results of the analysis demonstrated that there were 83 significantly differentially expressed miRNAs, which were divided into two categories: known miRNAs comprising 32 miRNAs (27 up-regulated, 5 down-regulated) and novel miRNAs comprising 51 miRNAs (9 up-regulated, 42 down-regulated) (Table S4).

**Figure 2:**
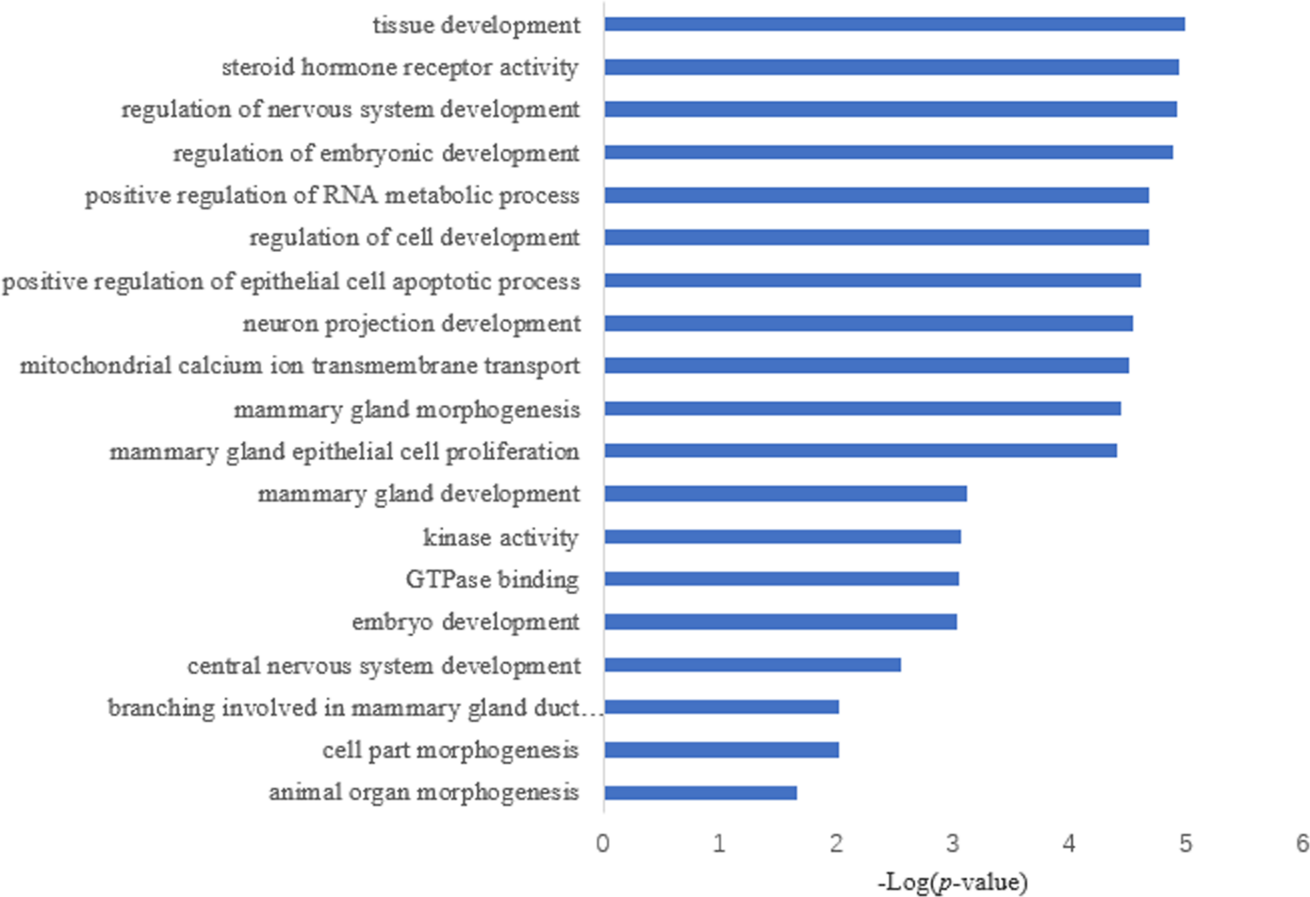
The differential miRNA expression profile in milk of Kazakh horse.

The effect of milk on the miRNA was also evidenced by a cluster analysis and a principle component analysis (PCA) (Fig. 2). Samples were clustered according to H and L groups (Fig. 2A). The first principal component (PC1) accounted for 41% of the total variance, and the second components (PC2) accounted for 19% of the total variance (Fig. 2B).

### Target gene prediction and gene functional annotation

**Figure 3:**
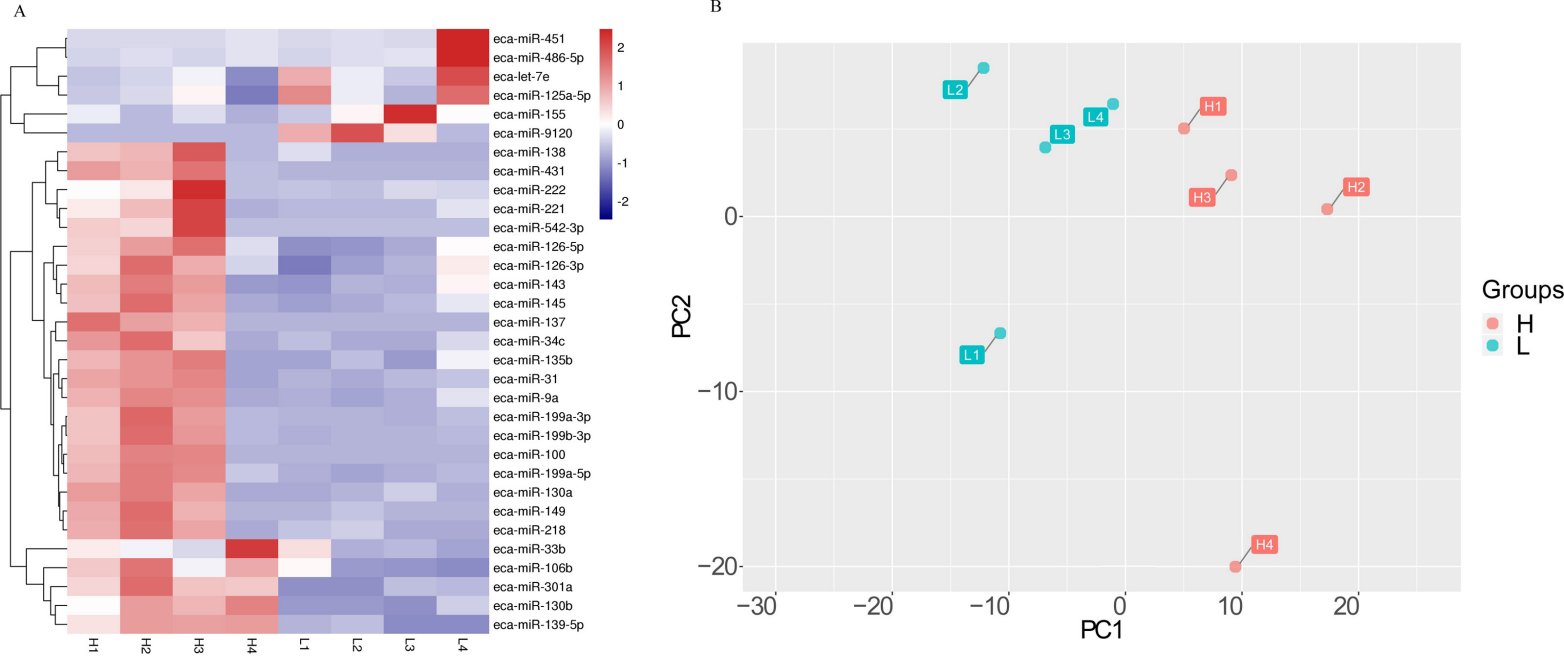
GO annotation analysis.

**Figure 4:**
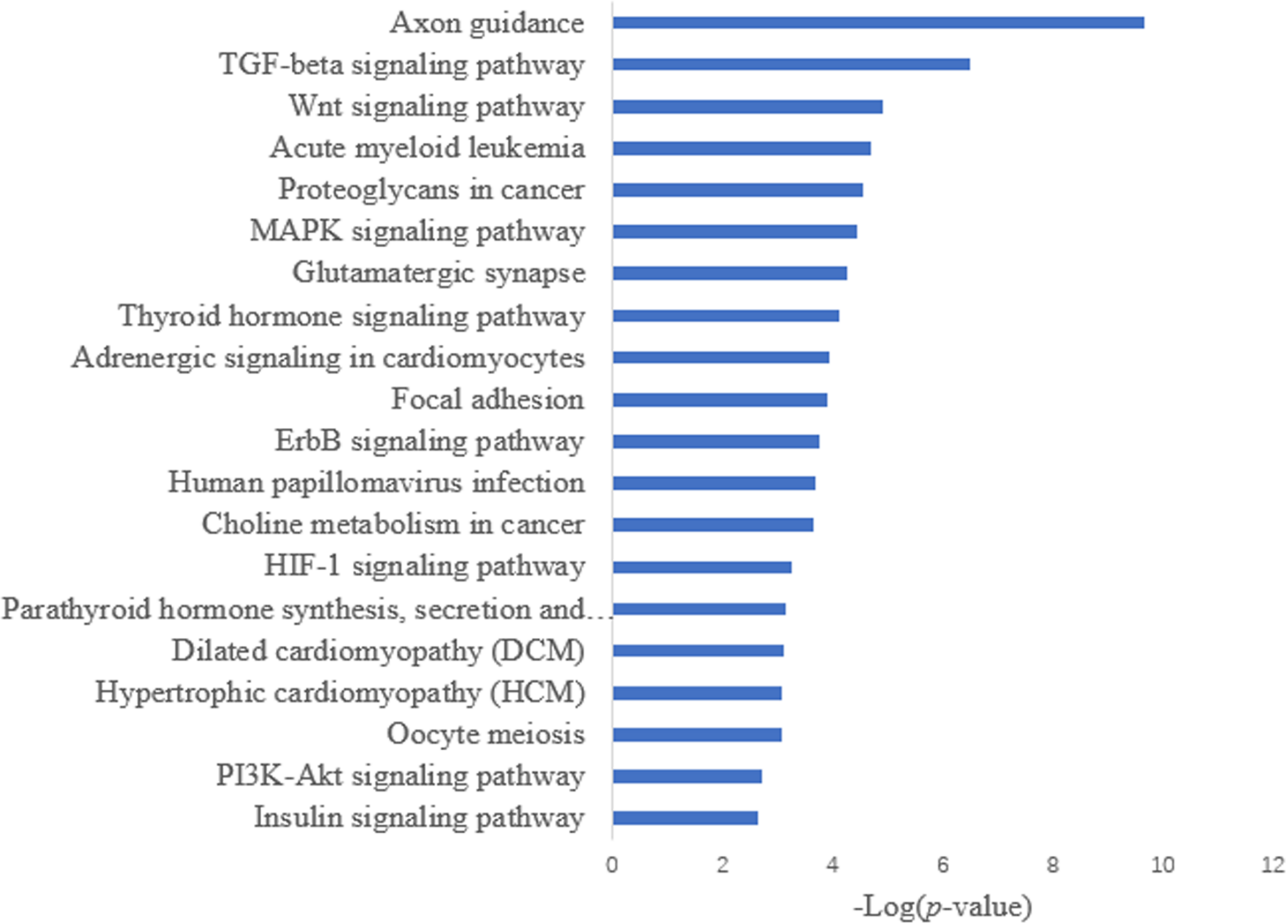
KEGG annotation analysis.

Target prediction is an important way to determine the functions of miRNAs. We predicted a total of 2,415 target genes among the differentially expressed miRNAs. GO gene function analysis showed the enrichment of some terms related to breast development, nervous system development, and kinase activity (Fig. 3). Results of the KEGG analysis showed that these target genes were involved mainly in the insulin signaling pathway, insulin secretion, breast cancer, oocyte division, and the thyroid hormone signaling pathway (Fig. 4).

### Validation of differentially expressed miRNAs

**Figure 5:**
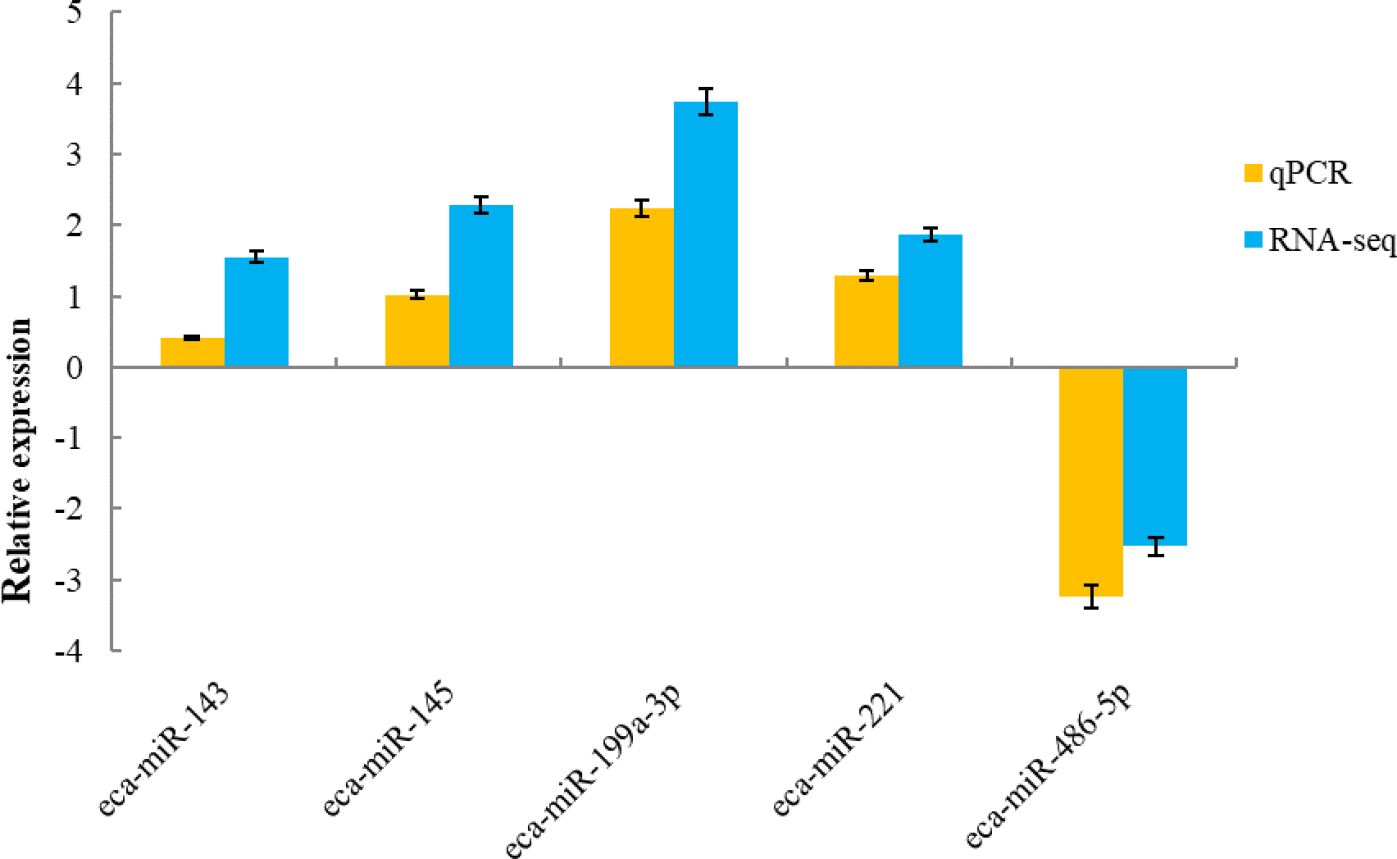
qRT-PCR validation of five miRNAs identified as differentially expressed by miRNA profiling.

To validate the sequencing data, five known miRNAs were selected for quantitative analysis. qPCR detection assays were used to confirm the expression of differentially expressed miRNAs in the H and L groups. The 5 miRNAs were verified using real-time PCR. The expression of miR-143, miR-145, miR-199a-3p and miR-221 were up-regulated, whereas miR-486-5p was down-regulated, which was in agreement with the RNA-sequencing results (Fig. 5).

## Discussion

Mammary gland development in dairy animals determines their lactation performance, which is important for the quality of dairy products (Silanikove et al., 2014).

We focused on miRNAs in the milk fat from two groups of Kazakh horses. A total of 83 miRNAs were found in the mammary gland. We reviewed the literature to determine the miRNAs related to mammary gland performance and development and found 5 miRNAs related to mammary gland development. The proliferation and apoptosis of mammary gland cells affects mammary gland development, milk secretion, and lactation (Capuco et al., 2001). miR-143 appeared to be expressed at relatively high levels in adipose or mammary gland tissues (Gu, Eleswarapu & Jiang, 2007). Our study showed that miR-143-induced mammary epithelial cell arrest in the G0/G1 phase delayed the progression of the cell cycle and inhibited cell proliferation (Ji et al., 2016). Furthermore, miR-143 inhibits the proliferation and promotes the apoptosis of mammary gland epithelial cells by targeting and regulating the expression of Ndfip1 (Ji et al., 2019). miR-145 promoted the formation of lipid droplets and triglycerides. Milk fat exists in the form of milk fat globules, and milk fat globules are found in the mammary epithelial cells (MEC) in the form of fat droplets before secretion. Triglycerides are the main component of fat droplets (Ducharme & Bickel, 2008). Therefore, it is thought that miR-145 has an important function in the synthesis of milk fat. Cao (2015) reported that the overexpression of miR-145 in goat mammary epithelial cells could significantly increase the content of lipid droplets in cells and promote the accumulation of intracellular triglycerides. miR-199a-3p affected milk yield by regulated the HK2 gene (Li et al., 2012). miR-221 plays a role in milk production (Wang et al., 2018) and has been found to be highly expressed in early lactation compared with fresh and dry periods, suggesting a role in the control of endothelial cell growth and proliferation (Wang et al., 2012). miR-221 may inhibit mammary gland epithelial cell proliferation by targeting STAT5a and IRS1, key genes in the PI3K-Akt/mTOR and JAK-STAT signaling pathways, respectively (Jiao et al., 2019). miRNA-486-5p was differentially regulated between the pregnant and non-pregnant subjects. It is thought that the targets of differentially regulated miRNAs during pregnancy participate in signaling pathways, such as the PI3/AKT signaling pathway, and other endocrine-based pathways (Loux et al., 2017), and that miRNA-486-5p may affect milk yield.

Sirtuin 1 (SIRT1) and insulin like growth factor (IGF) participate in the insulin signaling pathway that affected glucose utilization and glucose production during early lactation and can also participate in storing glycogen substrates during early lactation. Insulin increases the utilization of acetate in fat synthesis and reduced fat breakdown in adipose tissue. Insulin in dairy cows also regulates the synthesis of milk proteins (Wang, 2017).

The target gene of miRNA participated in the PI3K/AKT signaling pathway. PI3K/AKT signaling has been shown to play a role in trophectoderm migration (Lim & Song, 2016) and placental resource allocation (Sferruzzi-Perri et al., 2016). The PI3K and prolactin signaling pathways have been shown to increase pancreatic islet mass and sensitivity to glucose during pregnancy (Amaral et al., 2004), which may influence insulin receptor signaling and another affected pathway. The PITX2 gene regulated the Wnt/beta-catenin pathway to play a role in cell proliferation, differentiation, and growth and development (Basu & Roy, 2013).

## Conclusions

Five miRNAs were identified in relation to milk yield. We identified potential biomarkers for milk yield in early pregnancy and provided information about their targets genes and the affected pathways during pregnancy in the Kazakh horse.

##  Supplemental Information

10.7717/peerj.10796/supp-1 Supplemental Information 1 Milk yield, sequence and Differential miRNA

10.7717/peerj.10796/supp-2 Supplemental Information 2 Raw data

## Additional Information and Declarations

### DNA Deposition

The following information was supplied regarding the deposition of DNA sequences: miRNA sequences are available at NCBI: PRJNA684780.

### Data Availability

The following information was supplied regarding data availability: The raw measurements are available in the Supplemental Files.
