# Supplementary material for: Evaluation of key miRNAs during early pregnancy in Kazakh horse using RNA sequencing
Source: PeerJ. 2021 Feb 23;9:e10796. doi: 10.7717/peerj.10796 (PMC7908884; doi:10.7717/peerj.10796)
Supplement: Supplemental Information 1 [file peerj-09-10796-s001.zip › Supplemental Files/Table S1.docx]

**Table S1** The average daily milk production of H and L group in Kazakh horse

| Milk component | High group | Low group |
| --- | --- | --- |
| Number | 4 | 4 |
| Milk yield (kg/d) | 13.87±3.44 | 3.95±1.29 |
