# Supplementary material for: Evaluation of key miRNAs during early pregnancy in Kazakh horse using RNA sequencing
Source: PeerJ. 2021 Feb 23;9:e10796. doi: 10.7717/peerj.10796 (PMC7908884; doi:10.7717/peerj.10796)
Supplement: Supplemental Information 1 [file peerj-09-10796-s001.zip › Supplemental Files/Table S2.docx]

**Table S2** **Primer sequence of seven miRNAs**

| **Primer Name** | **Sequence(5'to3')** |
| --- | --- |
| U6（horse）(endogenous reference) | GCTTCGGCAGCACATATACTAA |
| eca-miR-26a（endogenous reference） | TTCAAGTAATCCAGGATAGGCTA |
| eca-miR-143 | TGAGATGAAGCACTGTAGCTCAA |
| eca-miR-145 | CAGTTTTCCCAGGAATCCCTAA |
| eca-miR-199a-3p | ACAGTAGTCTGCACATTGGTTAA |
| eca-miR-221 | GCTACATTGTCTGCTGGGTTTC |
| eca-miR-486-5p | GTACTGAGCTGCCCCGAGA |
