# Supplementary material for: Evaluation of key miRNAs during early pregnancy in Kazakh horse using RNA sequencing
Source: PeerJ. 2021 Feb 23;9:e10796. doi: 10.7717/peerj.10796 (PMC7908884; doi:10.7717/peerj.10796)
Supplement: Supplemental Information 1 [file peerj-09-10796-s001.zip › Supplemental Files/Table S3.docx]

**Table S3** **QC results of reads**

| Sample.ID | Total.Reads | Trimmed.Adaptor | Trimmed.N | QualityPercent | Trimmed.PolyA/T | Trimmed.Length | Clean.Data | Percentage |
| --- | --- | --- | --- | --- | --- | --- | --- | --- |
| L1 | 36699449 | 179893 | 3331 | 2472 | 5527 | 12964487 | 23543739 | 64% |
| L2 | 43485569 | 122651 | 6793 | 5122 | 4266 | 12782955 | 30563782 | 70% |
| L3 | 46154969 | 216849 | 6559 | 8210 | 4211 | 12749246 | 33169894 | 71% |
| L4 | 34676651 | 115074 | 4200 | 3332 | 2372 | 9609002 | 24942671 | 71% |
| H1 | 28025537 | 257392 | 7767 | 4265 | 5733 | 5131867 | 22618513 | 80% |
| H2 | 27361219 | 108860 | 6577 | 2523 | 3955 | 8027918 | 19211386 | 70% |
| H3 | 29331228 | 104884 | 9603 | 7510 | 4312 | 8829087 | 20375830 | 69% |
| H4 | 27732956 | 235103 | 10085 | 3439 | 4394 | 6917628 | 20562306 | 74% |
