# Supplementary material for: Evaluation of key miRNAs during early pregnancy in Kazakh horse using RNA sequencing
Source: PeerJ. 2021 Feb 23;9:e10796. doi: 10.7717/peerj.10796 (PMC7908884; doi:10.7717/peerj.10796)
Supplement: Supplemental Information 1 [file peerj-09-10796-s001.zip › Supplemental Files/Table S4.docx]

**Table S4** **Differentially expressed miRNAs in Kazakh horse**

| **miRNA** | **Log2(Fold Chang)** | **FDR** | **Up-/down-regulated** |
| --- | --- | --- | --- |
| eca-let-7e | -1.365669318 | 0.005141895 | down |
| eca-miR-100 | 7.206200388 | 4.80E-20 | up |
| eca-miR-106b | 1.133025419 | 0.048995219 | up |
| eca-miR-125a-5p | -1.051007966 | 0.037029851 | down |
| eca-miR-126-3p | 1.700439718 | 0.006201231 | up |
| eca-miR-126-5p | 1.959358016 | 0.003808963 | up |
| eca-miR-130a | 3.03562391 | 0.000123441 | up |
| eca-miR-130b | 2.415037499 | 0.002575803 | up |
| eca-miR-135b | 1.662965013 | 0.014476824 | up |
| eca-miR-137 | Inf | 7.84E-05 | up |
| eca-miR-138 | 3.321928095 | 0.000907065 | up |
| eca-miR-139-5p | 2.584962501 | 0.012587364 | up |
| eca-miR-143 | 1.557284049 | 0.001310479 | up |
| eca-miR-145 | 2.27085391 | 0.000348211 | up |
| eca-miR-149 | Inf | 6.95E-05 | up |
| eca-miR-155 | -1.078263549 | 0.001215752 | down |
| eca-miR-199a-3p | 3.738208761 | 5.08E-09 | up |
| eca-miR-199a-5p | 4 | 1.45E-08 | up |
| eca-miR-199b-3p | 3.559995585 | 2.70E-08 | up |
| eca-miR-218 | 2.584962501 | 0.013068381 | up |
| eca-miR-221 | 1.870162562 | 0.000550022 | up |
| eca-miR-222 | 1.5360529 | 0.044902728 | up |
| eca-miR-301a | 2.247927513 | 0.005697555 | up |
| eca-miR-31 | 3 | 6.51E-06 | up |
| eca-miR-33b | 1.098355072 | 0.028749378 | up |
| eca-miR-34c | 3 | 0.00331396 | up |
| eca-miR-431 | Inf | 3.44E-05 | up |
| eca-miR-451 | -5.033423002 | 3.56E-11 | down |
| eca-miR-486-5p | -2.520256811 | 0.000164538 | down |
| eca-miR-542-3p | Inf | 0.0001595 | up |
| eca-miR-9a | 2.350497247 | 0.000120899 | up |
| eca-miR-9120 | Inf | 0.036721623 | up |
| 1_2652 | -1.248710098 | 0.016414361 | down |
| 10_20023 | -1.930737338 | 0.003441525 | down |
| 10_20139 | -1.009297745 | 0.042793864 | down |
| 10_20830 | -2.047887329 | 0.000116679 | down |
| 11_21695 | -1.572334892 | 0.003302188 | down |
| 11_22448 | -1.395928676 | 0.028187668 | down |
| 11_22769 | -3.247927513 | 0.000433074 | down |
| 12_23335 | Inf | 0.004685434 | up |
| 13_24161 | Inf | 0.035895508 | up |
| 13_24781 | -1.788203822 | 0.000793736 | down |
| 13_25203 | -2.031026896 | 0.000336334 | down |
| 14_25349 | -1.639410285 | 0.005650997 | down |
| 15_27941 | 2 | 0.04175356 | up |
| 15_28011 | -1.237649087 | 0.010065157 | down |
| 15_28082 | -1.656045599 | 0.006542871 | down |
| 15_28179 | Inf | 0.004733427 | up |
| 15_28180 | Inf | 0.019782924 | up |
| 16_29136 | -1.259087221 | 0.049085003 | down |
| 18_32817 | Inf | 0.014416401 | up |
| 19_33817 | -3.169925001 | 0.004354642 | down |
| 2_3943 | -2.584962501 | 0.000120867 | down |
| 2_4419_star | Inf | 0.0102274 | up |
| 20_34754 | -1.193718113 | 0.002312014 | down |
| 22_36582 | -1.522840789 | 0.006593323 | down |
| 22_37005 | -2.428843299 | 0.00023508 | down |
| 22_37284 | -1.004374016 | 0.031310693 | down |
| 28_42996 | -1.528928466 | 0.005551707 | down |
| 28_43361 | -1.847996907 | 0.016575852 | down |
| 29_43746 | -1.529253068 | 0.007705318 | down |
| 29_43833 | -2.807354922 | 0.017461136 | down |
| 3_6639 | -2.874469118 | 0.000778312 | down |
| 3_6699 | -2.30718151 | 4.83E-05 | down |
| 3_7283 | -2.321928095 | 0.011476543 | down |
| 3_8412 | -3.280107919 | 1.10E-06 | down |
| 4_9875 | -1.699750258 | 0.000634243 | down |
| 5_10443 | -2.222392421 | 0.003806055 | down |
| 5_10518 | -1.700439718 | 0.004689035 | down |
| 5_11297 | -1.847996907 | 0.0021277 | down |
| 6_12840 | -1.906890596 | 0.020879896 | down |
| 6_13574 | -1.736965594 | 0.017726156 | down |
| 7_15178 | -2.459431619 | 0.009037713 | down |
| 8_16933 | -1.222915421 | 0.001873175 | down |
| 8_17968 | -1.277228287 | 0.035834441 | down |
| 8_18191 | -1.584962501 | 0.022568299 | down |
| 8_18347 | -1.678071905 | 0.044166606 | down |
| 9_19582 | Inf | 0.000768774 | up |
| 9_19673 | -1.556393349 | 0.002067433 | down |
| X_45569 | 1.338479271 | 0.01726904 | up |
| Un0054_47301 | -1.807354922 | 0.031752486 | down |
| Un0056_47291 | -1.868755467 | 0.001894942 | down |
| Un0270_48029 | -2.053111336 | 0.000698662 | down |
